# Salicylate and Procyanidin-Rich Stem Extracts of *Gaultheria procumbens* L. Inhibit Pro-Inflammatory Enzymes and Suppress Pro-Inflammatory and Pro-Oxidant Functions of Human Neutrophils *Ex Vivo*

**DOI:** 10.3390/ijms20071753

**Published:** 2019-04-09

**Authors:** Piotr Michel, Sebastian Granica, Anna Magiera, Karolina Rosińska, Małgorzata Jurek, Łukasz Poraj, Monika Anna Olszewska

**Affiliations:** 1 Department of Pharmacognosy, Faculty of Pharmacy, Medical University of Lodz, Muszynskiego 1 St., 90-151 Lodz, Poland; anna.magiera@umed.lodz.pl (A.M.); karolinaa.rosinskaa@gmail.com (K.R.); gosiaju11@gmail.com (M.J.); lukasz.poraj@gmail.com (Ł.P.); monika.olszewska@umed.lodz.pl (M.A.O.); 2 Department of Pharmacognosy and Molecular Basis of Phytotherapy, Faculty of Pharmacy, Warsaw Medical University, 1 Banacha St., 02-097 Warsaw, Poland; sgranica@wum.edu.pl

**Keywords:** *Gaultheria procumbens*, neutrophils, oxidative burst, anti-inflammatory activity, antioxidant activity, polyphenols, salicylates, procyanidins, interleukin 1β, elastase 2

## Abstract

Salicylate-rich plants are an attractive alternative to synthetic anti-inflammatory drugs due to a better safety profile and the advantage of complementary anti-inflammatory and antioxidant effects of the co-occurring non-salicylate phytochemicals. Here, the phytochemical value and biological effects in vitro and *ex vivo* of the stems of one of such plants, *Gaultheria procumbens* L., were evaluated. The best extrahent for effective recovery of the active stem molecules was established in comparative studies of five extracts. The UHPLC-PDA-ESI-MS^3^, HPLC-PDA, and UV-photometric assays revealed that the selected acetone extract (AE) accumulates a rich polyphenolic fraction (35 identified constituents; total content 427.2 mg/g dw), mainly flavanols (catechins and proanthocyanidins; 201.3 mg/g dw) and methyl salicylate glycosides (199.9 mg/g dw). The extract and its model components were effective cyclooxygenase-2, lipoxygenase, and hyaluronidase inhibitors; exhibited strong antioxidant capacity in six non-cellular in vitro models (AE and procyanidins); and also significantly and dose-dependently reduced the levels of reactive oxygen species (ROS), and the release of cytokines (IL-1β, IL-8, TNF-α) and proteinases (elastase-2, metalloproteinase-9) in human neutrophils stimulated *ex vivo* by lipopolysaccharide (LPS) and *N*-formyl-L-methionyl-L-leucyl-L-phenylalanine (*f*MLP). The cellular safety of AE was demonstrated by flow cytometry. The results support the application of the plant in traditional medicine and encourage the use of AE for development of new therapeutic agents.

## 1. Introduction

*Gaultheria* L. (Ericaceae) is a large plant genus of about 150 species distributed through the Americas, Asia and Australasia. Chemotaxonomically, representatives of the genus are characterized by the abundance of methyl salicylate 2-*O*-(6′-*O*-β-D-xylopyranosyl)-β-D-glucopyranoside (gaultherin, GT) and/or its structural analogues differing in glycosidic sugar components [1]. Natural salicylates are historically the first pure compounds used in anti-inflammatory therapies and model structures for the synthesis of aspirin (acetylsalicylic acid), the most widely used non-steroidal anti-inflammatory drug (NSAID) globally [2]. In comparison to NSAIDs, plant salicylates including GT offer a better safety profile in internal applications; as precursor and slow-release drugs they do not hydrolyze to the active salicylic acid molecule in the stomach and do not exert gastrointestinal toxicity and gastric ulcerogenic effects [3,4]. Salicylate-containing plants may thus be a valuable basis for the development of novel effective and safe anti-inflammatory agents in the form of both individual components and active extracts.

*Gaultheria procumbens* L. (American wintergreen, Ericaceae) is an aromatic, evergreen shrub native to north-eastern North America and cultivated worldwide in regions of temperate climate as an ornamental and medicinal plant. In traditional medicine, the aerial parts (stems and leaves) of *G. procumbens* and other *Gaultheria* species, as well as methyl salicylate-rich essential oils distilled from the plants, are used (both externally and internally) in the treatment of disorders connected with inflammation, pain, and/or infection, including rheumatoid arthritis, influenza, the common cold, tracheitis, pharyngitis, pleurisy, fever, prostatitis, swelling and muscular pain, and some skin and periodontal problems [1,3,5]. In contrast to *G. yunnanensis* (Franch.) Rehder and GT, the most popular Asian representative of the genus and its main salicylate component, the mechanisms and molecular vectors of anti-inflammatory action of *G. procumbens* are poorly recognized. The pure GT and its analogues isolated from *G. yunnanensis* have been demonstrated to exert anti-inflammatory effects by inhibiting the production of ROS and pro-inflammatory cytokines (TNF-α, IL-6, and IL-1β) and by suppressing the activation of the NF-κB signaling pathway in vitro [3,6,7]. The in vivo effects of GT have been described as similar to those of cyclooxygenase inhibitors from the group of NSAIDs [8]. Plant extracts, however, are usually multicomponent, and various constituents may influence their final activity. In comparison to pure compounds, complex polyphenolic matrices have the ability to affect multiple intracellular targets and produce complementary, additive, and/or synergistic anti-inflammatory effects [9]. According to our previous research, the leaf extracts of *G. procumbens* contain a variety of non-salicylate polyphenols (flavan-3-ols, A- and B-type procyanidins, flavonoids, and caffeoylquinic acids), which are strong antioxidants and exhibit relatively high inhibitory activity towards pro-inflammatory enzymes, lipoxygenase, and hyaluronidase [10,11]. The stems of *G. procumbens* have not been investigated to date, neither in terms of chemical composition nor biological activity, except in that the ability of this plant organ to accumulate similar levels of total salicylates to leaves has been documented [12]. The preliminary tests conducted prior to the present study indicated that, apart from salicylates, the stems may also accumulate high levels of procyanidins, thus making them a promising candidate for further research. Recently, numerous procyanidin oligomers have been shown to significantly modulate the pro-inflammatory response of human immune cells (neutrophils) [13] and murine macrophages [14].

Neutrophils are the most abundant circulating leukocytes in humans, forming the primary line of defense of the innate immune system. After mobilization from bone marrow, neutrophils migrate to the place of infection or injury, where they can actively drive phagocytosis and/or the inflammatory process. Activated neutrophils release many enzymes (matrix metalloproteinases, ELA-2, phospholipase A2, COX-2, lipoxygenases, etc.), chemical mediators (eicosanoids, cytokines, chemokines, etc.), and large amounts of ROS (O_2_^•‒^, ^•^OH, H_2_O_2_, HClO, etc.), which are responsible for their motility and physiological functions [15]. The exaggerated or unregulated prolonged activation of neutrophils can induce tissue damage and is believed to contribute to the pathogenesis of chronic human diseases with an inflammatory background, such as obstructive pulmonary disease, inflammatory bowel disease, atherosclerosis, rheumatoid arthritis, nephritis, psoriasis, and atopic dermatitis. [16]. As some of the pro-inflammatory enzymes and chemical mediators released by immune cells have become molecular targets for anti-inflammatory therapies [17], neutrophils constitute a valuable in vitro model for the evaluation of novel anti-inflammatory agents, including plant-based products [13,18,19].

Therefore, the aim of the present study was to evaluate for the first time the phytochemical profile (UHPLC-PDA-ESI-MS^3^, HPLC-PDA, and UV-spectrophotometry) of *G. procumbens* stem extracts and their effects on the pro-inflammatory and pro-oxidant functions (the release of ROS, IL-1β, IL-8, TNF-α, MMP-9, and ELA-2) of human neutrophils *ex vivo*. The best solvent for effective recovery of the active components and the most active extract for the cell-based assays were selected in non-cellular tests of pro-inflammatory enzyme inhibition (LOX, COX-2, and HYAL) and antioxidant activity (DPPH, TBARS, and FRAP), including scavenging for ROS typical of oxidative burst of neutrophils (O_2_^•‒^, ^•^OH, and H_2_O_2_). The extract effects were compared to those of the synthetic anti-inflammatory agents (DEX and IND) and model stem polyphenols (GT and PB2). Moreover, the cellular safety of the extracts was evaluated by flow cytometry in viability tests of neutrophils.

## 2. Results

### 2.1. Phytochemical Profiling of the Stem Extracts

**Figure 1:**
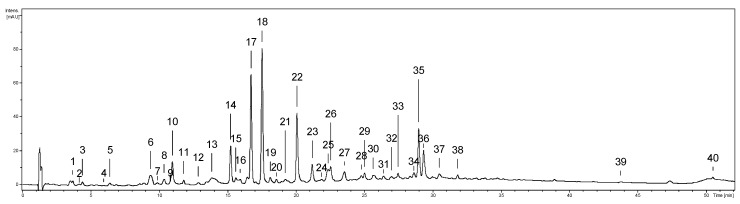
Representative UHPLC-PDA chromatogram of AE at 280 nm. The peak numbers refer to those implemented in Table S1.

The UHPLC-PDA-ESI-MS^3^ analysis of the *G. procumbens* stem extracts obtained with five solvents revealed the presence of 40 phenolic constituents (Figure 1, UHPLC peaks **1**–**40**), the structures of 35 of which were fully or tentatively characterized (Table S1) based on the comparison of their chromatographic and spectral data with the literature [10,20,21] and reference standards. The most structurally diversified group of the stem polyphenols were flavan-3-ols: (+)-catechin (**10**) and (‒)-epicatechin (**17**, ECA), and seventeen procyanidins including A-type dimers (**6**, **21**, **25**, **31**, **36**), B-type dimers (**7**, **8**, **13**, **14**, **20**, **24**, **28**), A-type trimers (**19**, **22**), and B-type trimers (**23**, **27**, **32**). They were accompanied by flavonoids including two aglycones, quercetin (**39**, QU) and kaempferol (**40**), and four quercetin monoglycosides (**33**, **34**, **35**, **37**) with the prevailing miquelianin (**35**, MQ). Phenolic acids were represented by simple hydroxybenzoic and hydroxycinnamic acids (**1**, **2**, **3**, **16**) and quinic acid pseudodepsides (**4**, **5**, **9**). Among the analytes, only one representative of lignans was found, lyoniresinol hexoside (**26**), and two methyl salicylate derivatives (**12, 18**) with the dominant gaultherin (**18**, GT). Most of the compounds were detected in all extracts (Table S1) but significant differences in their concentration levels and extraction yields were observed depending on the extraction solvent.

**Figure 2:**
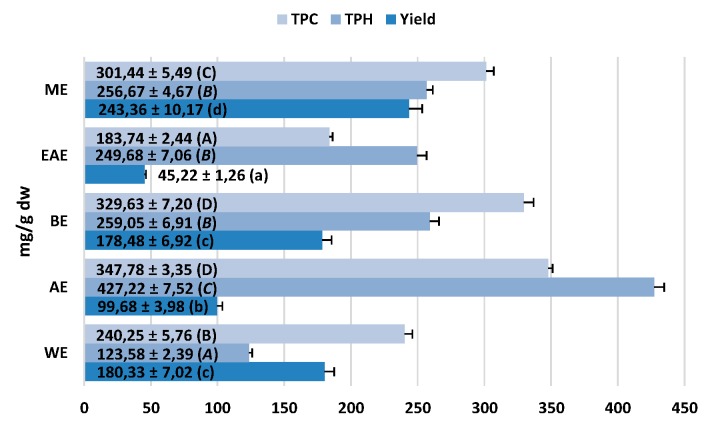
Total content of phenolic compounds (TPC, TPH) and extraction yields of the *G. procumbens* stem dry extracts. Data expressed as means ± SD (*n* = 3) per dw of the extract (TPC, TPH) and the plant material (Yield). For each parameter, different letters in parentheses indicate significant differences (*p* < 0.05).

**Figure 3:**
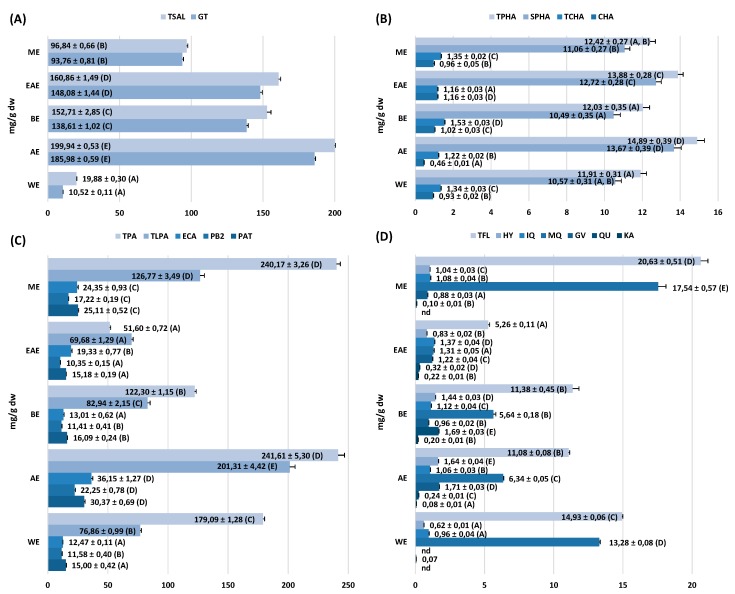
The quantitative data of individual compounds and groups of polyphenols in the stem dry extracts of *G. procumbens*: (**A**) salicylates; (**B**) phenolic acids; (**C**) flavan-3-ols and procyanidins; and (**D**) flavonoids. All data expressed as means ± SD (mg/g dw; *n* = 3 × 3 × 1) calculated per dw of the extracts. For each parameter, different superscripts (A–E) indicate significant differences (*p* < 0.05). Additional abbreviations: SPHA, total content of simple hydroxybenzoic and hydroxycinnamic acids; TCHA, total content of chlorogenic acid isomers; CHA, 5-*O*-caffeoylquinic acid (chlorogenic acid); HY, hyperoside; IQ, isoquercitrin; GV, guaijaverin; KA, kaempferol; nd—not detected.

The total phenolic content of the extracts varied in a wide range for both Folin–Ciocalteu-reactive substances (TPC, 183.7–347.8 mg GAE/g dw) and low molecular weight constituents detectable by RP-HPLC (TPH, 123.6–427.2 mg/g dw), and decreased as follows: AE ≥ BE > ME > WE > EAE, with different trend observed for the yield: ME > BE ≥ WE > AE > EAE (Figure 2). The primary constituents of the extracts were methyl salicylate glycosides (Figure 3A) and procyanidins (Figure 3C). The salicylate fraction (TSAL) was composed in 52.9–96.8% of GT and the peak levels of both were found in AE. The flavan-3-ols were determined as total procyanidins (TPA) and as low molecular weight derivatives (TLPA), which are known for the best bioavailability in humans. The most promising profile, i.e., high TPA levels with high relative contribution of TLPA, was revealed for AE, which was also the richest in ECA, PB2, and PAT as individual components (Figure 3C). Both flavonoids (TFL) and phenolic acids (TPHA) formed the minor fractions of the analyzed extracts (Figure 3B,D). In contrast to salicylates and procyanidins, flavonoids were concentrated mainly in ME. The dominant glycoside MQ constituted 24.9–88.9% of the TFL fraction. Phenolic acids were characterized by the lowest differences in total contents between the extracts, but the highest levels were found again in AE.

Based on the low extraction yield, the least promising extract (EAE) was excluded from further studies.

### 2.2. Antioxidant and Anti-Inflammatory Activity in Non-Cellular Models

The activity of four selected extracts (AE, ME, BE, and WE) and the model representatives of salicylates (GT) and procyanidins (PB2) were studied using six chemical tests of antioxidant activity (DPPH, FRAP, TBARS, as well as O_2_^•‒^, ^•^OH, and H_2_O_2_ scavenging assays) and three inhibition tests towards pro-inflammatory enzymes (LOX, HYAL, and COX-2). The extracts revealed concentration-dependent activity in all applied models, and AE was in general the most effective antioxidant and enzyme inhibitor (Table 1 and Table 2). However, in the ^•^OH and H_2_O_2_ scavenging assays, as well as in the TBARS and HYAL tests, the activity parameters of ME did not differ significantly from those of AE (*p* < 0.05). The responses in most of the models corresponded well with the levels of polyphenols (TPC, TPH, TLPA, and TSAL) (Table S2) and decreased in a similar order, i.e., AE ≥ ME > BE > WE. The exceptions were the scavenging tests of O_2_^•‒^ and ^•^OH, in which relatively stronger effects of WE were observed, and the COX-2 test, in which BE was relatively more effective. In the same conditions, the model constituents of the extracts exhibited complementary activity: PB2 was an extremely strong antioxidant (Table 1) and effective LOX inhibitor, GT suppressed the activity of COX-2, and both analytes were moderate HYAL inhibitors (Table 2).

In comparison to the positive standards—TX (a synthetic analogue of vitamin E) and IND (a non-steroidal anti-inflammatory drug)—the extracts exhibited significant activity, and AE was at most 1.5 times and 3 times less effective than the standards in the antioxidant and anti-inflammatory activity tests, respectively. Moreover, if activity parameters were recalculated to GAE (Table 2 and Table S3) using the TPC values, the obtained capacities of the phenolic fractions constituting the crude extracts were at least comparable to or up to 3 times higher than those of the standards.

### 2.3. Effect on the Viability and Pro-Inflammatory Functions of Human Neutrophils

**Figure 4:**
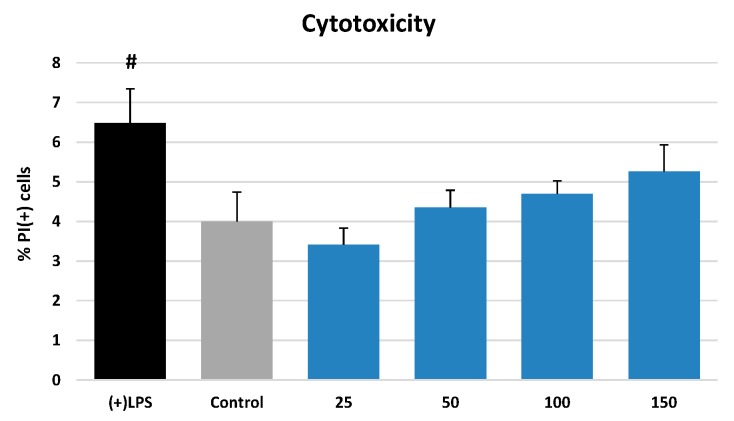
Effect of AE at 25–150 µg/mL on viability (membrane integrity) of neutrophils as indicated by propidium iodide positive [PI(+)] cells. Data expressed as means ± SD of three independent experiments performed with cells isolated from five independent donors. Statistical significance: #*p* < 0.05 compared with the non-stimulated control.

The most active extract (AE) in non-cellular models was further investigated in a model of human neutrophils. A standard flow cytometric probe with propidium iodide (PI) staining was used to demonstrate that AE did not influence the viability of the cells. In particular, at a wide concentration range of 25–150 µg/mL no significant reduction (*p* > 0.05) in membrane integrity was observed between neutrophils incubated with the extract [3.42–5.26% of PI(+) cells] and the untreated control [4.00 ± 0.74% of PI(+) cells] (Figure 4).

**Figure 5:**
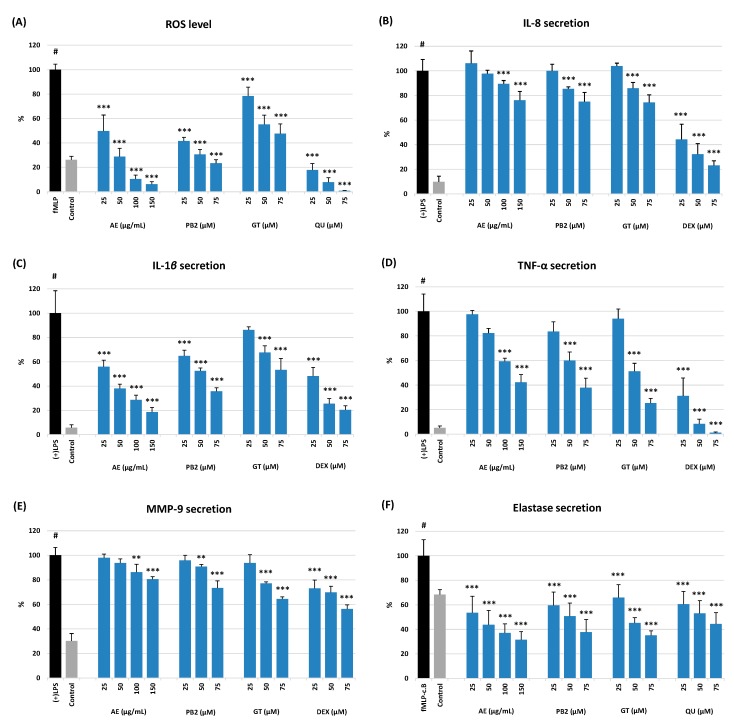
Effect of AE at 25, 50, 100, 150 µg/mL (6, 12, 24, 36 µg GAE/mL), model polyphenols (PB2 and GT), and positive controls (QU and DEX) on: (**A**) ROS level; and secretion of (**B**) IL-8; (**C**) IL-1β; (**D**) TNF-α; (**E**) MMP-9; and (**F**) ELA-2 by stimulated human neutrophils. Data expressed as means ± SD of three independent experiments performed with cells isolated from five independent donors. Statistical significance: #*p* < 0.001 compared to the non-stimulated control; ** *p* < 0.01, *** *p* < 0.001 compared to the stimulated control (+)LPS.

The anti-inflammatory activity of AE at 25–150 µg/mL (6.0–36.2 µg GAE/mL) was evaluated through the measurement of its impact on the ROS level and on the release of cytokines and proteinases by neutrophils stimulated by *f*MLP, *f*MLP-cytochalasin B, or LPS, depending on the test (Figure 5). The observed effects were concentration-dependent and the strongest for the levels of ROS, IL-1β, ELA-2, and to a lesser extent also for TNF-α. Incubation of the stimulated human neutrophils with AE resulted in a significant reduction of the ROS levels down to 49.7% at 25 µg/mL and 6.2% at 150 µg/mL in comparison to the levels produced by the *f*MLP-treated control cells (*p* < 0.001). The activity of AE was, however, weaker when compared with QU used as a positive control. The tested extract also exhibited a strong and statistically significant (*p* < 0.05) ability to inhibit the secretion of IL-1β and mitigate the ELA-2 release. In the presence of AE, the release of IL-1β decreased to 55.7% at 25 µg/mL (*p* < 0.05) and to 18.6% at 150 µg/mL (*p* < 0.001) in comparison to the LPS-stimulated cells. In addition, the effect of AE on IL-1β production at the highest tested concentration was similar to that of DEX at 30 µg/mL (75 µM; *p* > 0.05). The impact of the extract on the ELA-2 secretion did not differ from that of QU (*p* > 0.05) at the whole concentration range investigated, and the enzyme release decreased up to 31.6% at 150 µg/mL of AE in comparison with the *f*MLP-stimulated neutrophils (*p* < 0.001). The effects on TNF-α, IL-8, and MMP-9 secretion were less pronounced and statistically significant only at the elevated concentration levels (100–150 µg/mL). Nevertheless, at these concentrations the release of TNF-α was still noticeably reduced (up to 42.3%) in comparison to the LPS-stimulated cells. Both salicylates and procyanidins seem to be responsible for the observed effects of AE, as the model compounds GT and PB2 exhibited a similar activity profile and significantly influenced the levels of ROS, IL-1β, and ELA-2 in stimulated neutrophils at all tested concentrations (25–75 µM).

## 3. Discussion

The stems, leaves, and aerial parts (stems and leaves) of *G. procumbens* are widely used as anti-inflammatory, antipyretic, and analgesic agents [1,5]. The accumulated knowledge on GT and other salicylic acid analogues suggests that GT-rich *Gaultheria* plants might be a valuable basis for the development of new effective non-steroidal anti-inflammatory drugs [3,5,6,7,8]. The ethnomedicinal sources recommend the application of the plant materials in the form of traditional infusions and tinctures [1]. According to the overall recommendation of modern phytotherapy, however, standardized dry extracts might offer some advantage as they contain concentrated active constituents and provide higher therapeutic efficiency than traditional herbal products. Hence, in the present work, we evaluated the value of *G. procumbens* stem dry extracts as anti-inflammatory agents in complementary non-cellular and cellular in vitro models; we also optimized the extraction solvent for effective recovery of their active components. The solvents tested were methanol–water (75:25, *v/v*) and water which correspond to the traditional forms of herbal preparations, as well as acetone, ethyl acetate, and *n*-butanol, often reported as the best solvents for selective extraction of polyphenols from different matrices [13], including the leaves of *G. procumbens* [10]. Similar qualitative profiles and strong variations in the concentration levels of individual components were observed between the extracts (Figure 2 and Figure 3). In accordance with the previous findings on the leaves [10,11,20], five main fractions of active constituents were detected in the stems, including flavanols and procyanidins, salicylates, flavonoids, quinic acid pseudodepsides, and simple phenolic acids. In comparison with the leaves, however, the stems were distinguished by a noticeable diversity of procyanidins (17 vs. 5 compounds), low variation of flavonoids (6 vs. 10 compounds), and quinic acid pseudodepsides (3 vs. 7 compounds), and the presence of lignans (Table S1, Figure 1). Moreover, the crude methanol–water extract (the only one appropriate for the comparison) from the stems (ME) accumulated higher contents of polyphenols (301.4 vs. 270.7 mg GAE/g) and procyanidins (126.8 vs. 93.8 mg/g for TLPA), and similar levels of salicylates (96.8 vs. 98.9 mg/g for TSAL) as the leaves [10]. It might suggest that stems significantly contribute to the biological effects of aerial parts of *G. procumbens* reported by traditional medicine and may be especially advantageous for further application.

The present work is the first detailed study on the stems of *G. procumbens*, and on *Gaultheria* stems in general. The few previous reports focused only on selected aspects of the stem profiles. For example, Cong et al. [22] isolated from the stems of *G. fragrantissima* several polyphenols, including ECA, GT, MQ, and hyperoside, observed now also in *G. procumbens* (Table S1, Figure 1), as well as salicylic acid, quercetin 3-*O*-*β*-D-galacturonopyranoside, and quercetin 3-*O*-(2”-*O*-*β*-D-galacturono-pyranosyl)-*β*-D-glucopyranoside, which seem to be species-specific. Moreover, the presence of aryltetralin lignans, derivatives of isolariciresinol and lyoniresinol, were earlier detected in the stems of five *Gaultheria* species (at the levels of 1.4–2.2 mg/g dw): *G. griffithiana*, *G. tetramera*, *G. leucocarpa var. cumingiana*, *G. fragrantissima*, and *G. yunnanensis* [1]. In the stem extracts of *G. procumbens,* this group was represented by only one compound, i.e., lyoniresinol hexoside (peak 26, Table S1, Figure 1), found at the level up to 1.5 ± 0.05 mg/g dw in AE (results not shown), well within the range observed for other representatives of the genus. Low relative contents in comparison to other polyphenols, and the reports of Gao et al. [23] on the inactivity of *Gaultheria* lignans in a cellular model of inflammation (LPS-stimulated RAW 246.7 macrophages), suggest that their impact on the anti-inflammatory activity of *G. procumbens* stems is negligible.

With the abundant presence in the extracts (Figure 3), flavan-3-ol derivatives (including procyanidins) and salicylates were expected to influence the observed activity the most. A significant body of experimental evidence documents the anti-inflammatory effects of GT, ECA, and oligomeric procyanidins, both in vitro and in vivo. For instance, GT, isolated from the stems and leaves of *G. yunnanensis*, exhibited an antinociceptive effect, similar to that of aspirin, on acetic acid-induced abdominal contractions and inhibited croton oil-induced ear edema in a mouse model [3]. The GT-rich fraction (containing 80% of salicylates) of the same plant material showed significant analgesic effects in rats by reducing the number of writhings and strechings induced by the acetic acid, mitigating the time of paw licking forced by formalin when compared to aspirin, and inhibiting the carrageen-induced paw edema at a comparable level as IND [8]. It was also found that GT significantly and dose-dependently inhibits the secretion of TNF-α, IL-6, and IL-1β; suppresses the accumulation of NO; and reduces the ROS level in RAW264.7 murine macrophages stimulated by LPS [6]. Similarly, cinnamtannin B1, a trimeric A-type procyanidin structurally related to PAT [24], was reported to significantly decrease the intracellular ROS level and increase the activity of endogenous antioxidant enzymes, i.e., superoxide dismutase, catalase, and glutathione peroxidase, in a model of human hepatocytes [25]. Recently, numerous flavanol monomers, including ECA, procyanidin dimers, PB2, and higher procyanidin oligomers were showed to strongly influence the functions of human neutrophils *ex vivo*, reducing the ROS levels and downregulating the secretion of IL-8 and MIP-1β [13]. Moreover, procyanidin-rich fractions have often been proven to lower the levels of pro-inflammatory cytokines (such as IL-8, IL-6, and TNF-α) in vivo, for example, in a murine model of acute renal injury [26].

In the present study, the stem extracts of *G. procumbens* were found to contain high levels of salicylates and procyanidins, both in terms of the total contents (TSAL, TPA, and TLPA) and individual constituents (GT, ECA, PB2, and PAT). To confirm that these components might determine the anti-inflammatory activity of the stems and to establish the solvent for their effective recovery, four of the extracts exhibiting the highest extraction yield were screened for pro-inflammatory enzyme inhibition in non-cellular models. As the inflammatory process is closely linked to oxidative stress [27], the antioxidant activity of the extracts was also evaluated. The correlation studies confirmed that both fractions are co-responsible for the observed activity (Table S2). On the other hand, the analysis of the model compounds GT and PB2 indicated that the activity of both fractions is complementary: Procyanidins act as strong direct antioxidants (free radical scavengers, reductors of transition metal ions, and inhibitors of lipid peroxidation) and LOX inhibitors; salicylates are mainly COX-2 inhibitors; and both fractions influence the activity of HYAL with similar effectiveness. In consequence, AE, which accumulated the peak levels of salicylates and procyanidins (Figure 3), also presented the peak activity parameters in all tests (Table 1 and Table 2).

LOX, COX-2 and HYAL play the key role in mediator-implicated inflammation and are important targets of anti-inflammatory therapies. LOX and COX-2 are inducible enzymes catalyzing the incorporation of dioxygen molecules into polyunsaturated fatty acids (arachidonic acid), responsible for the release of chemokines and ROS, such as leukotrienes, prostaglandins, and O_2_^•‒^, at the site of inflammation [28,29]. The third enzyme, HYAL, breaks down hyaluronan, a chief polysaccharide component of the extracellular matrix, and increases the tissue permeability and the spread of pro-inflammatory mediators [30]. As AE was found to inhibit the three enzymes with an effectiveness (IC_50_ values) comparable to (HYAL) or, at most, 3-fold lower (LOX and COX-2) than that of synthetic anti-inflammatory drugs (IND and DEX, Table 2), it may be expected to reduce the inflammatory response in biological systems.

This hypothesis was verified in a model of human neutrophils obtained *ex vivo* from blood plasma buffy coats of healthy volunteers. The isolation procedure [31] allowed the obtaining of the neutrophil fractions in a steady state [32], characterized by a substantially high cell number, good viability, and the ability to produce, after stimulation, high levels of ROS and functionally significant pro-inflammatory cytokines and proteases (Figure 5). It was observed that AE significantly and in a dose-dependent manner reduced the levels of ROS and diminished the release of several tested cytokines and enzymes, especially IL-1β and ELA-2, from the stimulated neutrophils (Figure 5). It was also found that the extract did not deteriorate the viability of the cells (Figure 4).

The generation of ROS by immune cells in a process of oxidative burst is crucial for antimicrobial defense, but it may also lead to pathology via peroxidation of biomolecules (proteins and lipids) and activation of redox-sensitive signaling [15]. The primary reactive species released during the oxidative burst is O_2_^•‒^, that with the descendant ROS, such as ^•^OH, H_2_O_2_, and HClO, forms an aggressive mixture of oxidants [27]. We found that AE is a strong antioxidant able to significantly reduce the ROS level in neutrophils stimulated by the bacterial peptide *f*MLP (Figure 5), even at a low concentration level of 25 µg/mL (6 µg GAE/mL). The cell-based results corresponded with the high reducing capacity (FRAP) of the extract and PB2 observed in non-cellular tests, and their ability to directly scavenge ROS, including the species typical of neutrophils (Table 1). On the other hand, the relatively small difference between the antioxidant capacities of PB2 and GT in the cellular system, compared with that observed in chemical tests, suggests some indirect mechanisms would probably be involved in antioxidant protection, such as the activation of antioxidant enzymes, increased synthesis of lipoxins, or inhibition of the NF-*κ*B pathway, which have been documented in vivo for proanthocyanidins and salicylates [2,33]. The relatively high capacity of GT might also be connected with its metabolization to salicylic acid by cellular esterases [4]. Moreover, the protective activity of AE on cellular components (lipids) might be expected from its noticeable response in the TBARS test (Table 1). In vivo, the TBARS level, measuring the final stadium of the peroxidation process of serum lipoproteins, is a valuable biomarker of oxidative stress and progression of inflammation-related disorders [34].

Interleukin 1β is the primary pyrogenic cytokine released extracellularly by neutrophils and macrophages in response to inflammatory signals. IL-1β affects gene expression of many cytokines, including augmenting its own gene transcription; increasing the secretion of tissue-remodeling and pro-inflammatory enzymes, including MMPs, COX-2, and iNOS; and increasing the expression of leukocyte adhesion molecules and thrombogenic mediators. The blockage of IL-1β results in the retarded development of acute and chronic inflammatory disorders, such as rheumatoid arthritis, psoriasis, atopic dermatitis, pharyngitis, aphthous stomatitis, and atherosclerosis, among others [35]. A great number of these disorders are reported by traditional sources to be treated with *Gaultheria* preparations [1,5]. Indeed, in the present work we observed that AE is a potent inhibitor of the IL-1β release from the LPS-stimulated neutrophils (Figure 5). At 150 μg/mL (36 μg GAE/mL), the effectiveness of AE did not differ from that of DEX at 75 μM, which is promising for future application perspectives of the extract as an anti-inflammatory agent. The noticeable responses of GT and PB2 in the test indicated that both salicylates and procyanidins are co-responsible for the measured effects. It is in accordance with the previous reports on methyl salicylate glycosides as strong attenuators of the secretion of IL-1β from immune cells (macrophages) [6,7,36]. For instance, GT of *G. yunnanensis* was proved to decrease the release of IL-1β by LPS-stimulated RAW264.7 macrophages by 75.7% at 3 μg/mL [6]. In the case of procyanidins, the ability to reduce the production of IL-1β depends on the polymerization degree. Mao et al. [37] established that the inhibitory properties are typical for monomers through tetramers, while higher oligomers stimulate the production of the cytokine. With high relative ratio of low molecular weight compounds (TLPA/TPA = 0.83), procyanidins of AE thus appear to significantly determine the anti-IL-1β activity of the extract.

One of the most important tissue-remodeling enzymes released by neutrophils at sites of inflammation is ELA-2. It is a proteolytic enzyme degrading the extracellular matrix proteins, mainly elastin, as well as collagen and fibronectin. The elevated secretion of ELA-2 triggers numerous inflammatory disorders, especially respiratory diseases, but also rheumatoid arthritis, psoriasis, delayed wound healing, and oxidative stress-related premature skin aging with wrinkle formation [38]. As AE was found to significantly inhibit the secretion of ELA-2 from *f*MLP-cytochalasin B-stimulated neutrophils, and its inhibitory potency at 25 μg/mL (6 μg GAE/mL) did not differ from that of QU at 75 μM (Figure 5), the blockage of ELA-2 might represent an important mechanism of the anti-inflammatory activity of *G. procumbens* stems suggested by ethnomedicine. Although there are no previous reports of *Gaultheria*-derived phytochemicals as ELA-2 inhibitors, our results, especially the activity parameters of the model GT and PB2, are in accordance with the respective findings on salicylic acid [39] and oligomeric procyanidins [40].

## 4. Materials and Methods

### 4.1. Plant Material

Stems of *Gaultheria procumbens* L. were collected in October 2017 in the gardening center of Ericaceae plants, Gospodarstwo Szkolkarskie Jan Cieplucha (54^○^44′N, 19^○^18′E), Konstantynow Lodzki, Poland, where the plants grew in an open area. The seeds for the planting were imported from the William J. Beal Botanical Garden (Michigan State University, East Lansing, MI, USA), and authenticated by Piotr Banaszczak, Head of the Arboretum, Forestry Experimental Station of Warsaw University of Life Sciences (SGGW) in Rogow, Poland. The voucher specimen was deposited in the herbarium of the Department of Pharmacognosy, Medical University of Lodz, Poland, with the number KFG/HB/18001-GPRO-STEMS. Samples of the plant material were air-dried under normal conditions, powdered with an electric grinder, and sieved through a 0.315-mm sieve.

### 4.2. Preparation of Extracts

Five samples of the powdered stems (100 g each) were refluxed independently with different solvents: methanol-water (75:25, *v/v*), ethyl acetate, *n*-butanol, acetone, and water (3 times, 300 mL × 2 h each time). The combined extracts of each type were evaporated at 40 °C (*in vacuo*) to give five extracts: the dry stem methanol extract (ME), ethyl acetate extract (EAE), *n*-butanol extract (BE), acetone extract (AE), and water extract (WE). The remaining water residue was removed from the ME and WE by lyophilization using an Alpha 1-2/LD Plus freeze dryer (Christ, Osterode am Harz, Germany). The extraction procedure was repeated three times to establish the extraction yield for each extract (Figure 2). All quantitative results were calculated per dry weight (dw) of the extracts.

### 4.3. Phytochemical Profiling

The qualitative UHPLC-PDA-ESI-MS^3^ analysis was carried out according to Michel et al. [10]. The TPC and TPA were quantified by the Folin–Ciocalteu and *n*-butanol-HCl methods, respectively, as described previously [41]. Results were expressed as GAE and CYE, respectively.

The quantitative HPLC analyses were carried out on a HPLC VWR-Hitachi LaChrom Elite System (Hitachi, Tokyo, Japan) equipped with a quaternary pump, a photodiode array detector, an autosampler, and a thermostated column compartment with a C18 Ascentis Express column (2.7 μm, 75 mm × 4.6 mm i.d.), guarded by a C18 Ascentis C18 Supelguard guard column (3 μm, 20 mm × 4 mm i.d.; both from Supelco, Sigma-Aldrich, Seelze, Germany/St. Louis, MO, USA). Samples of the tested extracts (1–5 mg) were dissolved in 10 mL of methanol–water (70:30, *v/v*), filtered through a polytetrafluoroethylene (PTFE) syringe filter (25 mm, 0.2 µm, Ahlstrom, Helsinki, Finland), and the filtrate was directly injected (5 µL) into the HPLC system. The elution system consisted of solvent A (water-85% orthophosphoric acid, 100:0.5, *v/w*, pH 2.0) and solvent B (acetonitrile) with the elution profile as follows: 0–1 min, 6% B (*v/v*); 1–8.5 min, 6–14% B; 8.5–15 min, 14–16% B; 15–23 min, 16–50% B; 23–24 min, 50% B; 24–25 min, 50–6% B; 25–30 min, 6% B (equilibration). All solvents (Avantor Performance Materials, Gliwice, Poland) were of HPLC-grade purity. The flow rate was 1.4 mL/min, and the column was maintained at 18 °C. The phenolic analytes were quantified as equivalents of HPLC-pure external standards (Sigma-Aldrich, Seelze; Biopurify Phytochemicals, Chengdu, China): hydroxybenzoic acids as protocatechuic or *p*-hydroxybenzoic acids; caffeoylquinic acid isomers as chlorogenic acid (5-*O*-caffeoylquinic acid); hydroxycinnamic acid derivatives as *p*-coumaric or caffeic acids; dimeric and trimeric procyanidins as PB2 and procyanidin C1, respectively; methyl salicylate glycosides as GT; and flavonoid monoglycosides as MQ, depending on the PDA spectra. Apart from these reference compounds, the authentic standards of (+)-catechin, ECA, PB2, QU, and kaempferol (Phytolab, Vestenbergsgreuth, Germany) were also used for assessment of the corresponding peaks.

### 4.4. Anti-Inflammatory Activity in Non-Cellular Models

The ability of the extracts to inhibit LOX and HYAL was examined as described by Matczak et al. [42], while their inhibitory effect on COX-2 was evaluated by ELISA test following the instructions of the manufacturer (Cayman Chemical, Ann Arbor, MI, USA). All other reagents and standards, including IND, DEX and QU used as positive controls, were purchased from Sigma-Aldrich (Seelze). Prior to the assays, the analytes were dissolved in monosodium phosphate buffer (pH = 7.0) with 0.01% BSA, sodium borate buffer (pH = 9.0), or ELISA buffer and diluted to the final concentrations of 50-900 µg/mL, 2.5-50.0 µg/mL, and 50-1000 µg/mL for LOX, HYAL, and COX-2 tests, respectively. The exact concentration range for each analyte was established individually to cover 10–90% of the range of its activity. The results were expressed as IC_50_ values calculated from the eight-point concentration–inhibition curves.

### 4.5. Antioxidant Activity in Non-Cellular Models

The DPPH free-radical scavenging activity was determined as previously described [41] and expressed as normalized SC_50_ values. The FRAP was determined according to Olszewska et al. [41], expressed in µmol of ferrous ions (Fe^2+^) produced by 1g of the dry extract, which was calculated from the eight-point calibration curve of ferrous sulfate. The ability of the extracts to inhibit the AAPH-induced peroxidation of linoleic acid (LA) was determined according to Matczak et al. [42] and expressed as IC_50_ values. The O_2_^•‒^ scavenging capacity was determined according to Michel et al. [10] and expressed as SC_50_ values. To evaluate whether extracts affected the O_2_^•‒^ generation by direct interaction with xanthine oxidase, the enzyme activity was determined by monitoring the uric acid formation [10] and no inhibitory effect was observed. The ^•^OH and the H_2_O_2_ scavenging capacities were determined according to Fu et al. [43] and Fernando and Soysa [44], respectively, and expressed as SC_50_ values. The SC_50_, and IC_50_ parameters were calculated from the eight-point concentration-scavenging and concentration-inhibition curves. Prior to the analyses, the analytes were dissolved in methanol–water (75:25, *v/v*) or PBS, and diluted to the final concentrations of 0.8–350.0 µg/mL, 0.8–19.5 µg/mL, 0.5–450.0 µg/mL, 1.5–700.0 µg/mL, 15.0–700.0 µg/mL, and 2.5–700.0 µg/mL for DPPH, FRAP, TBARS, O_2_^•‒^, ^•^OH, and H_2_O_2_ methods, respectively. The exact concentration range for each analyte was established individually to cover 10–90% of the range of its activity. In all tests, QU and TX were used as positive controls. All reagents and standards were purchased from Sigma-Aldrich (Seelze). All tests were performed using 96-well plates and monitored using a microplate reader SPECTROstar Nano (BMG Labtech GmbH, Ortenberg, Germany). The samples were incubated in a constant temperature using a BD 23 incubator (Binder, Tuttlingen, Germany).

### 4.6. Antioxidant and Anti-Inflammatory Effects in Cellular Model

#### 4.6.1. Isolation of Human Neutrophils

The buffy coat fractions, being a by-product of blood fractionation for transfusions, were obtained from the Warsaw Blood Donation Centre, where they were collected from adult human donors (18–35 years old). The donors were clinically recognized to be healthy, and routine laboratory tests showed all values to be within the normal ranges. The study conformed to the principles of the Declaration of Helsinki.

Neutrophils were isolated with a standard method of dextran sedimentation prior to hypotonic lysis of erythrocytes and to centrifugation in a Ficoll Hypaque gradient [31]. The purity of the neutrophil fraction was over 97%. After isolation, cells were suspended in a (Ca^2+^)-free Hanks’ balanced salt solution (HBSS), (Ca^2+^)-free phosphate buffered saline (PBS), or RPMI 1640 culture medium and maintained at 4 °C before use. The (Ca^2+^)-free PBS was purchased from Biomed (Lublin, Poland), the Ficoll Hypaque gradient from PAA Laboratories, GmbH (Pasching, Austria), and all other reagents and media from Sigma-Aldrich (Seelze).

#### 4.6.2. Assessment of ROS Production by Human Neutrophils

The ROS production by *f*MLP-stimulated neutrophils was determined using the luminol-dependent chemiluminescence test [13]. The extracts were dissolved in HBSS and tested at the final concentration of 25–150 µg/mL. Following isolation, cells were suspended in HBSS. The protocol started by adding 50 µL of the tested extract in HBSS, then 70 µL cell suspension (3.5 × 10^5^/mL), 50 µL luminol (400 µg/mL), and finally by 30 µL *f*MLP (9.9 µg/mL) in a 96-well plate. The changes in chemiluminescence were measured over a 40 min period at intervals of 2 min in a microplate reader (Synergy 4, BioTek, Winooski, VT, USA). The percentage of ROS production was calculated in comparison to the control without the investigated extracts. QU (25–75 µM) was used as a positive control. All reagents and standards were purchased from Sigma-Aldrich (Seelze).

#### 4.6.3. Evaluation of IL-8, IL-1β, TNF-α and MMP-9 Release

The suspension of neutrophils (940 µL; 3.5 × 10^5^/mL) in an RPMI 1640 culture medium with 10% FBS, 10 mM *N*-(2-hydroxyethyl)piperazine-*N*′-(2-ethanesulfonic acid) (HEPES), 1% Penicillin-Streptomycin, and 2 mM L-glutamine was pre-incubated in 96-well plates for 1 h at 37 °C with 5% CO_2_, with the presence or absence of the tested extracts (50 µL) at the final concentrations of 25–150 µg/mL. Prior to the analyses, the extracts were dissolved in an RPMI 1640 medium. The cell suspension was then stimulated with LPS (10 µL, 10 µg/mL) and incubated for 24 h. After incubation, the plates were centrifuged (2000 RPM; 10 min; 4 °C) and supernatants were collected. The release of cytokines (IL-8, IL-1β, and TNF-α) and MMP-9 by stimulated neutrophils was evaluated by ELISA tests following the instructions of the manufacturer (BD Biosciences, San Jose, CA, USA, or R&D Systems, Minneapolis, MN, USA) using a microplate reader (Synergy 4). The percentage of cytokine production and enzyme release was calculated in comparison to the control without the investigated extracts. DEX (25–75 μM) was used as a positive control. LPS from *Escherichia coli* was purchased from Merck Millipore (Billerica, MA, USA). All other reagents and media were purchased from Sigma-Aldrich (Seelze).

#### 4.6.4. Evaluation of ELA-2 Release

The ELA-2 secretion by *f*MLP-cytochalasin B-stimulated neutrophils was determined using *N*-succinyl-alanine-alanine-valine *p*-nitroanilide (SAAVNA) as a substrate according to the method of Piwowarski and Kiss [19] with some modifications. Briefly, 200 µL of the neutrophil suspension (4.0 × 10^6^/mL) in HBSS was pre-incubated in 96-well plates for 15 min at 37 °C with 5% CO_2_, with the presence or absence of the tested extracts (50 µL) dissolved in HBSS, and tested at a final concentration of 25–150 µg/mL. The cell culture was then stimulated with 50 µL of *f*MLP (5.6 µg/mL) and cytochalasin B (2.8 µg/mL) for 15 min. After incubation, the plates were stored for 3 min on ice, and then centrifuged (2000 RPM; 10 min; 4 °C). The assay protocol began by adding 50 µL of SAAVNA solution (1.9 mg/mL) to 100 µL of the immediately harvested supernatants in a new 96-well plate. The extent of the released *p*-nitrophenol was measured at 412 nm over a period of 300 min with 20 min intervals using a microplate reader (Synergy 4). The percentage of the ELA-2 release was calculated in comparison to the control without the investigated extracts. QU (25–75 µM) was used as a positive control. All reagents and media were purchased from Sigma-Aldrich (Seelze).

### 4.7. Neutrophils Viability Studies

The potential cytotoxicity of the extracts was evaluated by a standard flow cytometric probe using propidium iodide (PI) staining. Neutrophils were cultured in 96-well plates in an RPMI 1640 medium (as above) with the presence or absence of the tested extracts at a final concentration of 25–150 µg/mL. After 24 h of incubation, the neutrophils were harvested and centrifuged (1500 RPM, 10 min, 4 °C), washed once with 500 µL of cold (Ca^2+^)-free PBS, centrifuged again (the supernatant was discarded), re-suspended in 500 µL of PI solution (0.5 µg/mL), and incubated in room temperature for 15 min in the dark. After this time, the cells were analyzed by flow cytometry (BD FACSCalibur apparatus, BD Biosciences, San Jose, CA, USA) and 10.000 events were recorded per sample. Cells that displayed high permeability to PI were expressed as a percentage of PI(+) cells. Cells treated with Triton X-100 solution were used as a control [98.6% of PI(+) cells].

### 4.8. Statistical Analysis

The results were expressed as means ± standard deviation (SD) of replicate determinations. The statistics (calculation of SD, one-way analysis of variance, HSD Tukey tests, and linearity studies) were performed using the Statistica12Pl software for Windows (StatSoft Inc., Krakow, Poland), with *p* values less than 0.05 being regarded as significant.

## 5. Conclusions

The present study demonstrates that the stems of *G. procumbens* are a rich source of structurally diverse polyphenols, among which methyl salicylate glycosides and procyanidins might be considered as the vectors of biological activity. The best solvent for effective recovery of the active components from the stems is acetone. The corresponding dry extract (AE) exhibits significant and dose-dependent antioxidant and anti-inflammatory activity both in non-cellular in vitro models and in a model of human neutrophils *ex vivo*. The extract reveals promising potential as a direct inhibitor of three pro-inflammatory enzymes (HYAL, COX-2, and LOX) and a modulator of pro-oxidant and pro-inflammatory functions of neutrophils stimulated by LPS, *f*MLP, and *f*MLP-cytochalasin B; it primarily influences the ROS levels (generated during the oxidative burst) and the release of IL-1β and ELA-2. The observed effects support the traditional usage of the stems in the treatment of inflammation-related disorders, especially the external application of tinctures in pharyngitis, tracheitis, as well as skin and periodontal problems, among others. The obtained results might also partly support the internal application of the stem preparations in rheumatoid arthritis, influenza, common cold, and fever. However, as polyphenols usually undergo intensive metabolic changes when ingested orally, further research on this issue, as well as in vivo experiments, are required to verify the extent of their systemic activity.

## Figures and Tables

**Table 1:** Antioxidant activity of *G. procumbens* stem dry extracts in non-cellular models.

Analyte	DPPH	FRAP	TBARS	O_2_^•‒^	^•^OH	H_2_O_2_
	SC_50_ (µg/mL) ^a^	mmol Fe^2+^/g ^b^	IC_50_ (µg/mL) ^c^	SC_50_ (µg/mL) ^a^	SC_50_ (µg/mL) ^a^	SC_50_ (µg/mL) ^a^
**AE**	5.67 ± 0.28 ^D^	7.65 ± 0.18 ^E^	6.70 ± 0.13 ^D^	22.44 ± 1.01 ^C^	149.24 ± 4.42 ^C^	33.01 ± 0.46 ^C^
**ME**	6.42 ± 0.20 ^E^	6.01 ± 0.06 ^C^	7.15 ± 0.70 ^D^	26.54 ± 0.64 ^D^	152.79 ± 0.26 ^C^	34.79 ± 0.07 ^C^
**BE**	6.62 ± 0.33 ^E^	6.41 ± 0.05 ^D^	12.12 ± 2.88 ^E^	34.49 ± 2.33 ^E^	178.96 ± 3.98 ^E^	38.28 ± 0.25 ^D^
**WE**	8.90 ± 0.36 ^F^	5.45 ± 0.16 ^B^	15.50 ± 0.18 ^F^	25.48 ± 1.24 ^D^	153.66 ± 4.70 ^C^	56.41 ± 2.18 ^E^
**PB2**	2.37 ± 0.02 ^B^	29.57 ± 0.11 ^G^	2.54 ± 0.13 ^B^	3.62 ± 0.05 ^A^	121.65 ± 3.42 ^B^	15.05 ± 0.56 ^B^
**GT**	265.69 ± 11.28 ^G^	0.64 ± 0.04 ^A^	269.40 ± 15.17 ^G^	451.76 ± 14.16 ^G^	488.52 ± 9.69 ^F^	587.86 ± 14.08 ^F^
**QU**	1.65 ± 0.04 ^A^	47.09 ± 0.61 ^H^	1.78 ± 0.06 ^A^	7.58 ± 0.21 ^B^	42.48 ± 4.07 ^A^	7.52 ± 0.38 ^A^
**TX**	4.31 ± 0.06 ^C^	11.89 ± 0.25 ^F^	4.68 ± 0.24 ^C^	135.24 ± 1.01 ^F^	165.45 ± 2.99 ^D^	15.87 ± 0.33 ^B^

^a^ Scavenging efficiency (amount of antioxidant needed to decrease the initial concentration of the oxidant by 50%) expressed in μg of the dry extract or standard/mL of the reaction solution; ^b^ values expressed per g of the dry extract or standard; ^c^ inhibition concentration (amount of antioxidant needed to decrease linoleic acid peroxidation and formation of TBARS by 50%). Results presented as mean values ± SD (*n* = 3). For each parameter, different capital letters given in parentheses (A–G) indicate significant differences (*p* < 0.05).

**Table 2:** Anti-inflammatory activity of *G. procumbens* stem dry extracts in non-cellular models.

Analyte	HYAL		LOX		COX-2	
	IC_50_ (µg/mL) ^a^	IC_50_ (μg GAE/mL; µM) ^b^	IC_50_ (mg/mL) ^a^	IC_50_ (mg GAE/mL; mM) ^b^	IC_50_ (mg/mL) ^a^	IC_50_ (mg GAE/mL; mM) ^b^
**AE**	11.67 ± 1.16 ^A, B^	4.06	0.29 ± 0.01 ^D^	0.10	0.38 ± 0.01 ^C^	0.13
**ME**	10.26 ± 0.86 ^A^	3.09	0.32 ± 0.01 ^E^	0.10	0.47 ± 0.01 ^E^	0.14
**BE**	15.09 ± 1.31 ^C^	4.97	0.38 ± 0.02 ^F^	0.12	0.44 ± 0.01 ^D^	0.14
**WE**	19.11 ± 1.28 ^D^	4.59	0.37 ± 0.01 ^F^	0.09	0.82 ± 0.03 ^G^	0.20
**PB2**	21.65 ± 1.03 ^E^	37.42 ± 1.78 ^A^	0.17 ± 0.01 ^C^	0.29 ± 0.03 ^A, B^	0.83 ± 0.04 ^G^	1.43 ± 0.06 ^D^
**GT**	28.58 ± 1.28 ^F^	64.02 ± 2.87 ^B^	0.56 ± 0.02 ^G^	1.26 ± 0.04 ^C^	0.35 ± 0.02 ^B^	0.78 ± 0.03 ^B^
**IND**	12.77 ± 1.91 ^B^	35.69 ± 5.34 ^A^	0.09 ± 0.01 ^A^	0.26 ± 0.01 ^A^	0.18 ± 0.01 ^A^	0.50 ± 0.02 ^A^
**DEX**	14.18 ± 1.05 ^C^	36.13 ± 2.68 ^A^	0.12 ± 0.01 ^B^	0.30 ± 0.02 ^B^	0.51 ± 0.02 ^F^	1.29 ± 0.04 ^C^
**QU**	30.78 ± 1.84 ^F^	101.84 ± 6.09 ^C^	0.09 ± 0.01 ^A^	0.30 ± 0.02 ^B^	0.47 ± 0.02 ^E^	1.56 ± 0.05 ^E^

^a,b^ Inhibition concentration (amount of analyte needed for 50% inhibition of enzyme activity) expressed as follows: ^a^ in μg/mg of the dry extract or standard/mL of the enzyme solution; ^b^ in μg/mg of phenolics/mL of the enzyme solution (values obtained by converting the original IC_50_ values using the TPC levels), or µM/mM of the standard. Results are presented as mean values ± SD (*n* = 3). For each parameter, different capital letters given in parentheses (A–G) indicate significant differences (*p* < 0.05).

## Supplementary Materials

Supplementary materials can be found at https://www.mdpi.com/1422-0067/20/7/1753/s1; Table S1: Phenolic analytes detected in *G. procumbens* stem dry extracts by UHPLC-PDA-ESI-MS^3^; Table S2: Correlation (*r*) coefficients and probability (*p*) values of linear relationships between antioxidant and anti-inflammatory activity parameters and phenolic contents of *G. procumbens* stem dry extracts; Table S3: Antioxidant activity parameters of *G. procumbens* stem dry extracts expressed in phenolic units.

## Abbreviations

AE	acetone extract
BE	*n*-butanol extract
COX-2	cyclooxygenase 2
CYE	cyanidin chloride equivalents
DEX	dexamethasone
DPPH	2,2-diphenyl-1-picrylhydrazyl
dw	dry weight
ECA	(–)-epicatechin
ELA-2	elastase 2
*f*MLP	*N*-formyl-L-methionyl-L-leucyl-L-phenylalanine
FRAP	ferric reducing antioxidant power
GAE	gallic acid equivalents
GT	methyl salicylate 2-*O*-(6′-*O*-β-D-xylopyranosyl)-β-D-glucopyranoside (gaultherin)
H_2_O_2_	hydrogen peroxide
HYAL	hyaluronidase
IND	indomethacin
IL	interleukin
LOX	lipoxygenase
LPS	bacterial lipopolysaccharide
ME	methanol extract
MMP-9	matrix metalloproteinase 9
MQ	quercetin 3-*O*-β-D-glucuronopyranoside (miquelianin)
O_2_^•‒^	superoxide anion
^•^OH	hydroxyl radical
PAT	procyanidin A-type trimer
PB2	procyanidin B2
QU	quercetin
ROS	reactive oxygen species
TBARS	thiobarbituric acid-reactive substances
TFL	total content of flavonoids
TLPA	total content of low-molecular weight flavanols and proanthocyanidins (HPLC)
TNF-α	tumour necrosis factor α
TPA	total proanthocyanidin content (*n*-butanol/HCl assay)
TPC	total phenolic content (Folin-Ciocalteu assay)
TPH	total phenolic content (HPLC)
TPHA	total content of phenolic acids
TSAL	total content of salicylates
TX	(±)-6-hydroxy-2,2,7,8-tetramethylchroman-2-carboxylic acid (Trolox^®^)
WE	water extract
