# Supplementary material for: Salicylate and Procyanidin-Rich Stem Extracts of Gaultheria procumbens L. Inhibit Pro-Inflammatory Enzymes and Suppress Pro-Inflammatory and Pro-Oxidant Functions of Human Neutrophils Ex Vivo
Source: Int J Mol Sci. 2019 Apr 9;20(7):1753. doi: 10.3390/ijms20071753 (PMC6479601; doi:10.3390/ijms20071753)
Supplement: Supplementary file 1 [file ijms-20-01753-s001.pdf]

International Journal of
Molecular Sciences

MDPI

Article

Salicylate and Procyanidin-Rich Stem Extracts of *Gaultheria procumbens* L. Inhibit Pro-Inflammatory Enzymes and Suppress Pro-Inflammatory and Pro-Oxidant Functions of Human Neutrophils *Ex Vivo*

Piotr Michel [1,*], Sebastian Granica [2], Anna Magiera [1], Karolina Rosińska [1], Małgorzata Jurek [1], Łukasz Poraj [1], and Monika Anna Olszewska [1]

[1] Department of Pharmacognosy, Faculty of Pharmacy, Medical University of Lodz, Muszynskiego 1 St., 90-151 Lodz, Poland; E-Mail: anna.magiera@umed.lodz.pl (A.M.); karolinaa.rosinskaa@gmail.com (K.R.); gosiaju11@gmail.com (M.J.); lukasz.poraj@gmail.com (Ł.P.); monika.olszewska@umed.lodz.pl (M.A.O.)

[2] Department of Pharmacognosy and Molecular Basis of Phytotherapy, Faculty of Pharmacy, Warsaw Medical University, 1 Banacha St., Warsaw 02-097, Poland; sgranica@wum.edu.pl (S.G.)

* Correspondence: piotr.michel@umed.lodz.pl (P.M.)

Supplementary Materials

Table S1. Phenolic analytes detected in *G. procumbens* stem dry extracts by UHPLC-PDA-ESI-MS^3.

Table S2. Correlation (*r*) coefficients and probability (*p*) values of linear relationships between antioxidant and anti-inflammatory activity parameters and phenolic contents of *G. procumbens* stem dry extracts.

Table S3. Antioxidant activity of *G. procumbens* stem dry extracts expressed in phenolic units.

Table S1. Phenolic analytes detected in *G. procumbens* stem dry extracts by UHPLC-PDA-ESI-MS^3.

Peak	Analyte	Rt (min)	UV λ_{max} (nm)	$[M–H]^-$ m/z	MS^2 (% relative abundance)	MS^3 (% relative abundance)	Extract
1	protocatechuic acid hexoside	3.9	259, 293	315	225 (15); 153 (100); 107 (15)		AE, ME, BE, WE
2	protocatechuic acid *	4.2	259, 293	153			All
3	caffeic acid derivative	4.7	325	305	219 (100); 179 (73); 125 (15)		All
4	3-*O*-caffeoylquinic acid (neochlorogenic acid) *	6.0	325	353	191 (100); 179 (55)		AE, ME, BE, WE
5	3-*O*-*p*-coumaroylquinic acid derivative	6.5	310	371	353 (22); 325 (63); 281 (12); 163 (100)		All
6	procyanidin A-type dimer	9.5	280	575	499 (100); 451 (17); 407 (22); 289 (23)		All
7	procyanidin B-type dimer	10.1	280	577	**425 (100)**; 407 (57); 289 (19)	407 (100); 273 (2)	AE, ME, BE, EAE
8	procyanidin B-type dimer	10.5	280	577	**425 (100)**; 407 (56); 289 (18)	407 (100); 273 (6)	All
9	5-*O*-caffeoylquinic acid (chlorogenic acid, **CHA**) *	10.9	325	353	191 (100); 179 (5)		All
10	(+)-catechin *	11.1	280	289	245 (100); 205 (40)		All
11	unknown compound	11.9	296	385	357 (6); 287 (5); 223 (100)		All
12	methyl salicylate derivative	13.0	285	653 [a]	607 (100); 575 (10)		AE, ME, BE, WE
13	procyanidin B-type dimer	14.1	280	577	**425 (100)**; 407 (59); 289 (29)	407 (100); 273 (8)	AE, ME, WE
14	procyanidin B2 (**PB2**) *	15.3	280	577	**425 (100)**; 407 (53); 289 (14)	407 (100); 273 (7)	All
15	unknown compound	15.7	280	461	415 (100); 167 (9)		All
16	protocatechuic acid derivative	16.1	259, 293	481	463 (7); 345 (72); 327 (100); 165 (28); 153 (62)		All
17	(–)-epicatechin (**ECA**) *	16.8	280	289	245 (100); 205 (24)		All
18	gaultherin (**GT**) *	17.7	285	491 [a]	445 (15); 413 (3); 293 (72); 233 (3); 149 (5)		All
19	procyanidin A-type trimer	18.3	280	863	**711 (100)**; 573 (19); 559 (15); 451 (30); 411 (34); 289 (13)	693 (76); 559 (69); 541 (24); 407 (12)	All
20	procyanidin B-type dimer	18.7	280	577	**425 (100)**; 407 (40); 289 (52)	407 (100); 273 (10)	All
21	procyanidin A-type dimer	19.5	280	575	499 (20); 491 (22); 451 (25); **425 (100)**; 407 (96); 289 (36)	407 (100); 273 (6)	AE, ME, BE, EAE
22	procyanidin A-type trimer (**PAT**)	20.3	280	863	**711 (100)**; 693 (14); 573 (25); 559 (16); 451 (23); 411 (24); 289 (7)	693 (64); 559 (100); 541 (27); 463 (10); 407 (16); 285 (4)	All

Table S1. *Cont.*

Peak	Analyte	Rt (min)	UV λ_{max} (nm)	$[M–H]^-$ m/z	MS^2 (% relative abundance)	MS^3 (% relative abundance)	Extract
23	procyanidin B-type trimer	21.3	280	865	847 (25); 739 (73); **713 (41)**; 695 (77); 577 (98); 451 (21); 287 (17)	695 (100); 575 (22); 561 (23); 407 (22); 243 (13)	All
24	procyanidin B-type dimer	22.1	280	577	**425 (100)**; 407 (44); 289 (22)	407 (100); 273 (11)	All
25	procyanidin A-type dimer	22.6	280	575	499 (69); 491 (24); 451 (22); **425 (100)**; 407 (79); 289 (25)	407 (100); 273 (9)	All
26	lyoniresinol hexoside	22.8	280	581	565 (16); **419 (100)**; 401 (12); 373 (15)	404 (100); 373 (16)	All
27	procyanidin B-type trimer	23.6	280	865	847 (21); 739 (47); **713 (51)**; 695 (92); 577 (99); 451 (52); 287 (54)	695 (100); 575 (24); 561 (26); 407 (25); 243 (10)	All
28	procyanidin B-type dimer	25.0	280	577	**425 (100)**; 407 (19); 287 (51)	407 (100); 273 (17)	AE, ME, WE
29	unknown compound	25.1	278	597	577 (10); **553 (100)**	536 (2); 419 (100); 389 (24); 233 (13)	All
30	unknown compound	25.8	278	567	**521 (100)**	359 (100); 344 (96); 329 (28)	AE, ME, BE
31	procyanidin A-type dimer	26.5	280	575	499 (27); 451 (22); **425 (100)**; 411 (57); 289 (7)	407 (100); 273 (7)	AE, ME, BE, WE
32	procyanidin B-type trimer	27.1	280	865	577 (82); 407 (26); 289 (18)		AE, ME, BE, WE
33	quercetin 3-*O*-β-D-galactopyranoside (hyperoside, **HY**) *	27.6	254, 353	463	**301 (100)**	273 (38); 255 (20); 179 (100); 151 (64)	All
34	quercetin 3-*O*-β-D-glucopyranoside (isoquercitrin, **IQ**) *	28.6	256, 353	463	**301 (100)**	273 (54); 255 (22); 179 (100); 151 (76)	All
35	quercetin 3-*O*-β-D-glucuronopyranoside (miquelianin, **MQ**) *	29.1	256, 356	477	**301 (100)**	273 (29); 257 (19); 179 (100); 151 (58)	All
36	procyanidin A-type dimer	29.5	280	575	449 (41); **423 (100)**; 289 (19)	405 (8); 285 (100); 257 (5)	All
37	quercetin 3-*O*-α-L-arabinopyranoside (guaijaverin, **GV**) *	30.8	258, 356	433	**301 (100)**	273 (44); 255 (21); 179 (100); 151 (68)	AE, ME, BE, EAE
38	unknown compound	31.9	278	567	521 (100); 179 (33)		All
39	quercetin (**QU**) *	43.7	255, 364	301	273 (36); 255 (21); 179 (100); 151 (69)		All
40	kaempferol (**KA**) *	50.4	265, 364	285	267 (45); 257 (100); 229 (80); 163 (15); 151 (18)		AE, BE, EAE

Rt, retention times. UV λ_{max}, absorbance maxima in PDA spectra. $[M−H]^-$, pseudomolecular ions in MS spectra recorded in a negative ion mode. In bold – ions subjected to MS^3 fragmentation. Compounds marked with an asterisk (*) were identified with authentic standards. [a] $[M+HCOO]^-$.

International Journal of *Molecular Sciences*

MDPI

Table S2. Correlation (*r*) coefficients and probability (*p*) values of linear relationships between antioxidant and anti-inflammatory activity parameters and phenolic contents of *G. procumbens* stem dry extracts.

r (*p*) for:	Antioxidant activity						Anti-inflammatory activity		
	DPPH	FRAP	TBARS	$O_2^{\bullet-}$	$^{\bullet}OH$	H_2O_2	HYAL	LOX	COX-2
TPC	−0.9523 (0.048) *	0.8857 (0.114)	−0.7312 (0.269)	0.1236 (0.876)	0.2291 (0.771)	−0.9127 (0.087)	−0.6998 (0.300)	−0.4797 (0.520)	−0.9729 (0.027) *
TPH	−0.9171 (0.083)	0.9773 (0.023) *	−0.8302 (0.170)	−0.2958 (0.704)	−0.1750 (0.825)	−0.8491 (0.151)	−0.7303 (0.270)	−0.7638 (0.236)	−0.8661 (0.134)
TPA	−0.3806 (0.619)	0.3443 (0.656)	−0.7292 (0.271)	−0.3806 (0.619)	−0.8987 (0.101)	−0.3804 (0.620)	−0.6502 (0.350)	−0.9257 (0.074)	−0.2147 (0.785)
TLPA	−0.7476 (0.252)	0.8691 (0.131)	−0.8364 (0.164)	−0.6647 (0.335)	−0.5705 (0.429)	−0.6772 (0.323)	−0.7005 (0.299)	−0.9448 (0.055)	−0.6300 (0.370)
TPHA	−0.6748 (0.325)	0.9158 (0.084)	−0.6978 (0.302)	−0.6378 (0.362)	−0.4982 (0.502)	−0.5740 (0.426)	−0.5322 (0.468)	−0.8490 (0.151)	−0.5703 (0.430)
TSAL	−0.9161 (0.084)	0.9419 (0.058)	−0.6902 (0.310)	0.0383 (0.962)	0.1726 (0.827)	−0.8533 (0.147)	−0.6265 (0.374)	−0.5029 (0.497)	−0.9266 (0.073)
TFL	0.1608 (0.839)	−0.5707 (0.429)	−0.1858 (0.814)	−0.1645 (0.836)	−0.3802 (0.620)	0.0308 (0.969)	−0.3085 (0.691)	−0.1445 (0.855)	0.2200 (0.780)

Activity and concentration parameters according to Figs. 2 and 3. Asterisk mean significance of the estimated linear relationship (* $p < 0.05$) for four extracts (data points $n = 4$).

International Journal of
Molecular Sciences

MDPI

Table S3. Antioxidant activity of *G. procumbens* stem dry extracts expressed in phenolic units.

Analyte	DPPH	FRAP	TBARS	$O_2^{\bullet-}$	$^{\bullet}OH$	H_2O_2
	SC_{50} (μg GAE /mL) [a]	mmol Fe^{2+}/g GAE [b]	IC_{50} (μg GAE/mL) [c]	SC_{50} (μg GAE/mL) [a]	SC_{50} (μg GAE/mL) [a]	SC_{50} (μg GAE/mL) [a]
AE	1.97	21.99	2.33	7.80	51.90	11.48
ME	1.94	19.94	2.16	8.00	41.06	10.49
BE	2.18	19.45	3.99	11.37	58.99	12.62
WE	2.14	22.68	3.72	6.12	36.92	13.55

[a] Scavenging efficiency (amount of antioxidant needed to decrease the initial concentration of the oxidant by 50%) expressed in μg of phenolics/mL of the DPPH solution (values obtained by converting the original SC_{50} values using the TPC levels); [b] values expressed per g of phenolics (obtained by converting the original FRAP values using the TPC levels); [c] inhibition concentration (amount of antioxidant needed to decrease linoleic acid peroxidation and formation of TBARS by 50%) expressed in μg of phenolics/mL of the substrate solution (values obtained by converting the original IC_{50} values using the TPC levels). For original TPC, FRAP, SC_{50} and IC_{50} parameters see Table 1 and Fig. 2.
